# Feeling psychologically restrained: the effect of social exclusion on tonic immobility

**DOI:** 10.3402/ejpt.v5.22928

**Published:** 2014-03-13

**Authors:** Nora Mooren, Agnes van Minnen

**Affiliations:** 1 Behavioural Science Institute, Radboud University Nijmegen, Nijmegen, The Netherlands; 2 Overwaal, Centre for Anxiety Disorders Overwaal, Nijmegen, The Netherlands

**Keywords:** Tonic immobility, social exclusion, psychological restraint, fear, relational aggression, trauma, PTSD, bullying

## Abstract

**Background:**

A variety of studies have demonstrated posttraumatic stress disorder (PTSD) symptoms in victims of bullying. Because bullying with only relational aggression, such as social exclusion, does not involve physical aggression that could explain PTSD symptoms, it remains unclear why these relational aggression situations are also linked to PTSD symptoms.

**Objective:**

The aim of the present study was to investigate whether the fear-response tonic immobility (Ti) can occur during social exclusion. Since Ti, as an indicator of peritraumatic dissociation, is an important predictor of PTSD symptoms, we expected that the presence of Ti during social exclusion might contribute to possible explanations of PTSD symptoms in victims of relational aggression.

**Method:**

Social exclusion was manipulated by a virtual Cyberball game in which participants were excluded and included by virtual confederates. During the game, Ti was measured, both physiologically (heart rate) and psychologically (subjective symptoms). Also, the underlying concepts of Ti, high levels of fear and psychological restraint (threatened sense of control), were measured.

**Results:**

Excluded participants experienced higher levels of subjective and physiological Ti symptoms (lower heart rates) in comparison to social inclusion. Also, as expected, social exclusion resulted in higher levels of fear and psychological restraint in comparison to social inclusion.

**Conclusion:**

Social exclusion can evoke symptoms of Ti, fear, and psychological restraint, which might be important mechanisms to consider in explaining PTSD symptoms after relational forms of bullying in the absence of physical aggression.

**Limitations:**

The sample only contains healthy, female participants. Whether our results translate to bullying victims of relational aggression is therefore not known. Also, the physiological measurement of Ti (average heart rate) was rather limited and could be expanded in future studies.

There is a growing body of literature highlighting the existence of posttraumatic stress disorder (PTSD) symptoms in victims of bullying (Idsøe, Dyregrov, & Idsøe, 2012; Matthiesen & Einarsen, 2004; Mynard, Joseph, & Alexander, 2000). Bullying can be defined as “longstanding violence, physical or mental, conducted by an individual or a group and directed against an individual who is not able to defend himself in the actual situation” (Roland, 1989, p. 143). Bullying happens at different ages and in several settings (e.g., school and work) (MacDonald & Leary, 2005). Frequently, bullying consists of a combination of relational aggression and physical aggression (Olweus, 2013; Roland & Idsøe, 2001). Physical aggression involves behaviors such as physical attack and fighting (Olweus, 1991; Roland & Idsøe, 2001), whereas relational aggression is a type of aggression that “involves behaviors such as threatening to withdraw friendship in order to get one's own way or using social exclusion as a form of retaliation” (Crick, Bigbee, & Howes, 1996). Examples include social exclusion, cyber bullying, and spreading rumors (Crick & Grotpeter, 1995; Williams & Guerra, 2007). In school bullying that included both physical and relational aggression, it was found that 25–37% of the victims reported PTSD symptoms (Mynard et al., 2000; Rivers, 2004). In bullying among adults in the workplace, 40–63% of the victims experienced PTSD symptoms (Matthiesen & Einarsen, 2004; Tehrani, 2004). Remarkably, in this study by Matthiesen and Einarsen (2004) bullying only included relational and verbal aggression, such as insulting remarks, social exclusion, verbal abuse, and spreading rumors. As such, this study shows that relational aggression, although it does not meet the DSM-5 A-criterion of PTSD (American Psychiatric Association, 2000), is associated with PTSD symptoms.

In the present study, we examined processes during one specific form of relational aggression: social exclusion. The aim of the present study was not to argue that relational aggression can fulfill the DSM-5 A-criterion, but rather to investigate what underlying processes, that are also present during trauma's that meet the DSM-5 A-Criterion, could occur during situations of relational aggression and elicit PTSD symptoms. In order to investigate this question, we zoomed in on a specific fear-response, namely tonic immobility (Ti), a peritraumatic dissociative response, which is known as an important predictor of PTSD in victims of physical trauma (Ozer, Best, Lipsey, & Weiss, 2003; Rocha-Rego et al., 2009). Ti is observed as a last defense response in reaction to a predator, after fight or flight reactions have failed to enable escape from the situation. Ti has both physiological symptoms such as immobility and stiffness, bradycardia, fixed, unfocused eye gaze, parkinsonian-like tremors, and decreased pain perception, as well as subjective symptoms such as an inability to speak or move and feeling fearful, cold, and ashamed (Gallup, 1977). According to the fear hypothesis (Gallup, 1977), Ti is commonly hypothesized to occur exclusively during situations that involve physical aggression, as described in reports of rape and sexual abuse (also referred to as *rape paralysis*). However, it was shown that organisms in social isolation show more prolonged immobility reactions than organisms that were not socially isolated (Gallup, 1974). Gallup (1974) concluded that: “… social isolation … could also lend itself to an interpretation of immobility as being related to fear associated with separation from imprinted or familiar companions” (p. 840). Experimental studies on social exclusion have indeed observed several Ti responses such as analgesia (DeWall & Baumeister, 2006; Eisenberg, Liebermann, & Williams, 2003; MacDonald & Leary, 2005), feeling cold (Zhong & Leonardelli, 2008), and experiencing the inability to control the social situation (Williams, 2007; Williams, Cheung, & Choi, 2000; Williams et al., 2002).

According to the fear hypothesis (Gallup, 1977), Ti has two necessary conditions: fear and physical restraint. In bullying situations that involve physical aggression, both components are present and Ti could be a possible trauma response and, subsequently, be a possible predictor of PTSD symptoms (Ozer et al., 2003; Rocha-Rego et al., 2009). However, in situations that only involve relational aggression, the physical restraint component is not present. This brought up our main research question: whether Ti can occur during situations with high levels of fear and relational aggression but no physical aggression or restraint, in this case social exclusion. Our hypothesis was that Ti can occur because the subjective experience of restraint rather than the actual physical restraint might be most evident in events that do not involve physical aggression. In the current study, we refer to this form of restraint as *psychological restraint*, which involves the subjective feeling of being restrained by others with the power to influence social status, accompanied by a perceived inability to control the social situation. For example, Mynard et al. (2000) showed that PTSD symptoms were predicted by the belief that control lies with powerful others. Psychological restraint is related to a wider range of situations. For instance, feeling restrained is often interpreted as an urge to leave an aversive situation or environment (flight), but being unable to move away from this situation because of social rank factors (Gilbert, Allan, Brough, Melley, & Miles, 2002). As such, the psychological experience of feeling restrained can take place in events involving physical aggression (e.g., rape or abuse) or other circumstances (e.g., restrained by partner in relationship) (Marx, Forsyth, Gallup, Fusé, & Lexington, 2008).

Thus far, only the study by Roelofs, Hagenaars, and Stins (2010) purposefully examined the relationship between relational aggression, in this case social threat with no physical restraint, and Ti (freeze). It was found that viewing angry faces induced fear and physiological Ti-like symptoms such as significant reductions in body movement and decreased heart rate. However, immobility was only measured with physiological indicators in that study and their experimental condition was assumed to induce fear, but not psychological or physical restraint. To fill this gap, the present study aimed to measure Ti during social exclusion that involves both fear and psychological restraint. Given the fact that social exclusion more frequently happens online than some years ago (Slonje & Smith, 2008; Williams & Guerra, 2007), the present study used the Cyberball exclusion game to evoke social exclusion. This game was used because it has been repeatedly shown that both fear and the psychological feeling of threatened control are induced. That is, participants feel anxious during this game (Williams, 2007), they exhibit the fear component of Ti, and participants have a threatened sense of control (Williams et al., 2000; Williams & Zadro, 2001; Zadro, Williams, & Richardson, 2004), which is hypothesized to be the psychological restraint component.

The present study aimed to test the following questions. First, we questioned whether Ti can occur during social exclusion. That is, we tested whether participants showed more subjective and physiological symptoms of Ti during social exclusion compared to during social inclusion. Second, we explored the two conditions of Ti, so whether social exclusion indeed evoked increased levels of fear and psychological, as opposed to physical, restraint. It was expected that social exclusion enhanced fear and psychological restraint in comparison to social inclusion.

## Method

### Participants

A total of 6 male (*n*=6) and 56 female (*n*=56) undergraduate students volunteered as participants. Their age ranged from 18 to 25 years (*M*=19.95 years, SD=2.07). They received a credit for their participation. All participants were informed about the procedure by a written informed consent prior to the experiment, but were kept naive with respect to the hypotheses. The study was approved by the local ethics committee.

### Materials

#### Cyberball game

To induce feelings of social exclusion and social inclusion, we used the Cyberball game (Williams et al., 2000; Williams & Jarvis, 2006). This computer program consists of four players (including the participant) who are playing a ball-tossing game. The participants were informed that they will play a ball game with students from another university. In order to make the cover story more believable, the game was programmed to open in Internet Explorer and the experimenter received a fake phone call from the experimenter of the fake university. The participants were instructed to throw the ball to one of the three other players, as soon as they received the ball. In the present study, the behavior of the other players was already determined by the computer program. In the social inclusion condition, participants received the ball in one third of the tosses. In the social exclusion condition, the participants received the ball three times before they were excluded from the game. After these throws the participant never received the ball again. In both conditions, the game lasted 5 min. In response to an additional question of the Threatened Needs Scale, 71% of the players believed that they actually engaged in a ball game with real players.

### Control measures

#### Social exclusion manipulation check

Manipulation of social exclusion was checked by the examination of the perception of the inclusion status. Participants indicated to what extent (on a five-point Likert-type scale) they felt excluded and ignored. In addition, current positive and negative mood was measured with four bipolar items (“I felt sad” vs. “I felt happy”) on a five-point Likert-type scale. A final question asked participants to estimate the amount of throws they received during the game (Zadro, Boland, & Richardson, 2006; Zadro et al., 2004).

#### Threatened Needs Scale

This 12-item questionnaire was used to evaluate the feelings of social exclusion on primary social needs (Zadro et al., 2004, 2006). With a five-point Likert-type scale (1=*not at all*, 5=*extremely*), this scale measured four important needs of participants during the game: belongingness (five items) (“I felt I belonged to the group”), control (four items) (“I felt I was unable to influence the actions of others”), self-esteem (five items) (“I felt good about myself”), and meaningful existence (six items) (“I felt meaningless”). These four needs in our sample have acceptable internal consistencies: belonging (*α*=0.74), control (*α*=0.71), self-esteem (*α*=0.73), and meaningful existence (*α*=0.76). The combined subscales in this sample have good internal consistency (*α*=0.88).

### Experimental measures

#### Fear

##### State-anxiety (STAI-state)

We measured state-anxiety with the Spielberger State-Trait Anxiety Inventory (STAI; Spielberger, Gorsuch, Lushene, Vagg, & Jacobs, 1983). This inventory consists of 20 items using a scale ranging from 1 (*not at all*) to 4 (*very much*), where higher scores indicate more state-anxiety. The STAI has high internal consistency (*α*=0.90) and good test–retest reliability (*r*=0.70–0.76) (Spielberger et al., 1983).

###### Psychological restraint

Psychological restraint was derived from the control scale of the Threatened Needs Scale. This control scale consists of four items that measured the subjective feeling of threatened control (“I felt I was unable to influence the actions of others”), (“I felt I had control over the course of the game”), (“I felt I the other players decided everything”). This scale has acceptable internal consistency (*α*=0.71). Further, one item (“Please indicate to what extent you felt restraint during the game”) of the Tonic Immobility Scale (TIS) was used as an additional indicator of psychological restraint.

#### Tonic immobility

##### Tonic Immobility Scale

In order to measure subjective symptoms of Ti during the social exclusion game, we used the TIS, translated and adapted by De Kleine, Van Minnen, and Hagenaars (2009). This questionnaire is originally used to measure Ti during traumatic events. For this study, the sentence “… during the unpleasant or traumatic event,” was replaced by “… during the game.” Items (#19) were scored from 0 (*not at all*) to 6 (*extremely*); higher scores indicated more Ti. The revised version has high internal consistency in the current sample (*α*=0.80).

###### Heart rate

As an indication of physical symptoms of Ti, we measured average heart rate over the periods of inclusion or exclusion by means of the program emWave^®^ by Heart Math^®^. This program measures average heart rate and pulse by a clip electrode on the finger of the participant. The pulse was used to check whether the signal of the clip was accurate.

### Procedure

All participants were allocated to both the social inclusion and social exclusion condition. The sequence was counterbalanced and the measurements were the same across the two conditions. All participants were given an informed consent and a questionnaire about demographic descriptions. Subsequently, participants were instructed about the Cyberball game. The instruction was the same for both the social inclusion and social exclusion condition. They were told that this study tested the effects of practicing mental visualization on task performance. They were asked to mentally visualize the experience and create a complete mental picture of what might be going on if they were playing this game in real life. Each time the participants received the ball, they had to throw the ball to one of the three other players by clicking on one of the names of the other players. During the game, heart rate was measured with an electrode on the finger. After they finished the first game in the one condition (exclusion or inclusion) the participants completed the manipulation check measures, TNS, TIS, and the STAI-State. Next, the whole procedure with the other game condition was repeated. At the end of the experiment, participants were debriefed about the cover story.

### Statistical analyses and design

Statistical analyses were conducted using SPSS version 19.0. An *a priori* power analysis indicated that in order to achieve a power level (1-*b*) of 0.95, a sample size of 45 would be required. The manipulation checks and the TNS, TIS, heart rate, psychological restraint, and STAI-state were analyzed by a repeated measures analysis of variance (ANOVAs) with condition (exclusion or inclusion) as within-subject factor. Subsequently, we calculated the Pearson correlations between Ti and fear, heart rate and fear, and heart rate and psychological restraint. Alpha was set at 0.05. The internal consistencies were calculated for the measures TNS, the control scale, self-esteem scale, belongingness scale, meaningful existence scale, and the TIS and were based on the measurements of the exclusion condition.

## Results

Three participants were observed as outliers, indicated by *z* scores greater than 3. They were excluded from further analyses. Because the final sample only contained six male participants, the results could only be generalized to females. Therefore, all males were excluded from further analyses. The final sample included 53 female participants.

### Control measures

#### Social exclusion manipulation check

Participants in the exclusion condition rated on the perception of inclusion status that they felt more excluded and ignored than during social inclusion, *F*(1, 52)=273.30, *p*<0.001, *η* ^2^=0.840 (see Table 1 for descriptive statistics). After the social exclusion condition, participants rated more negative mood (“I felt, bad, sad, angry, unfriendly”), *F*(1, 52)=31.74, *p*<0.001, *η* ^2^=0.379, and less positive mood (“I felt, good, happy, pleasant, friendly”), *F*(1, 52)=53.10, *p*<0.001, *η* ^2^=0.505. Also, when participants were excluded they reported a smaller percentage of received throws than when they were included, *F*(1, 52)=146.22, *p*<0.001, *η* ^2^=0.738. An analysis with “order” as between-subject factor proved that there were no differences between groups, *F*(13, 39)=1.27, *p*=0.269, *η* ^2^=0.298.

**Table 1:** Descriptive statistics of control measures

	Social inclusion		Social exclusion	
		
	M	SD	M	SD
Feeling ignored and excluded	2.74	1.11	7.77	1.91
Estimated throws received (%)	32.98	16.89	6.09	3.48
Threatened belongingness	9.62	3.33	19.26	3.08
Threatened self-esteem	11.00	3.07	14.57	3.49
Threatened meaningful existence	13.94	4.10	22.34	3.90
Threatened control	10.75	3.20	17.21	2.45
Negative mood	6.08	2.34	8.68	3.17
Positive mood	14.96	2.55	12.17	2.92

#### Threatening Needs Scale

As expected, during social exclusion participants rated more threatened feelings of belongingness, *F*(1,52)=251.12, *p*<0.001, *η* ^2^=0.828, self-esteem, *F*(1,52)=60.92, *p*<0.001, *η* ^2^=539, control, *F*(1,52)=129.65, *p*<0.001, *η* ^2^=0.714, and meaningful existence, *F*(1,52)=131.40, *p*<0.001, *η* ^2^=0.716 (see Table 1 for descriptive statistics).

### Experimental measures

#### Fear

##### State-anxiety

During social exclusion participants reported more state-anxiety (*M*=36.02, SD=8.82) than during inclusion (*M*=32.96, SD=7.19), *F*(1, 52)=13.34, *p*<0.01, *η* ^2^=0.204.

#### Psychological restraint

As expected, during social exclusion participants were higher in threatened control (*M*=17.21, SD=2.45) than during social inclusion (*M*=10.75, SD=3.20), *F*(1,52)=129.65, *p*<0.001, *η* ^2^=0.714. Also, participants reported significantly more feelings of restraint during social exclusion (*M*=1.60, SD=1.66) than during social inclusion (*M*=0.83, SD=1.31), *F*(1,52)=14.56, *p*<0.001, *η* ^2^=0.219.

#### Tonic immobility

##### Subjective symptoms

It was found that participants experienced significantly more symptoms on the TIS during social exclusion (*M*=23.87, SD=14.67) than during social inclusion (*M=*16.83, SD*=*11.18), *F*(1, 52)=28.55, *p*<0.001, *η* ^2^=0.354. The subjective symptoms of Ti during social exclusion were positively correlated with subjective fear (*r*=0.419, *p*<0.01).

###### Heart rate

Heart rate was lower during social exclusion (*M*=59.81, SD=26.25) than during social inclusion (*M*=75.56, SD=11.35), *F*(1, 52)=22.36, *p*<0.001, *η* ^2^=0.301. No significant relationships were found between heart rate and fear or heart rate and psychological restraint during exclusion (all, *r*=<0.182, *p*=>0.837).

## Discussion

The general aim of this study was investigating whether the fear-response Ti could occur during social exclusion that involves fear but no physical restraint. In order to investigate this question, we aimed to test whether social exclusion evoked subjective and physiological symptoms of Ti. In terms of the manipulation, we found that participants felt more excluded and ignored than during inclusion. Also, participants experienced higher levels of negative mood and lower levels of positive mood after exclusion. Finally, participants estimated a lower number of throws received during social exclusion in comparison to inclusion. These findings support the assumption that the manipulation of social exclusion succeeded. In agreement with previous studies that induced social exclusion (e.g., Williams et al., 2000; Zadro et al., 2004), we also found that social exclusion threatened the four needs of belongingness. Excluded participants reported higher levels of feeling an outsider, feeling invisible and meaningless, and lower levels of self-esteem. Also participants felt more threatened in their ability to claim a role in the group and reported low levels of belongingness to the group during social exclusion. Our first research question was whether Ti can occur during social exclusion. That is, we investigated whether participants showed significantly more subjective and physiological symptoms of Ti during social exclusion in comparison to social inclusion. In line with our hypotheses, it was found that excluded participants experienced higher levels of subjective symptoms of Ti during social exclusion in comparison to social inclusion. As expected, regarding the physiological symptoms of Ti, we found that heart rate was lower during exclusion. This is in line with the study by Moor, Crone, and Van der Molen (2010) and the study by Roelofs et al. (2010). However, it should be noted that we measured the average heart rate during the game whereas, if we adopt the assumptions of Ratner (1976) and Schauer and Elbert (2010), heart rate should first be high (fight or flight) and subsequently decline during Ti. This might explain why average heart rate was presumably a limited measure for physiological Ti.

One explanation for the fact that symptoms of Ti can occur during social exclusion with no physical aggression is that, in contrast to earlier assumptions, not only the actual level of physical restraint might predict fear-responses such as Ti, but also the subjective, psychological level of restraint. This was examined in our second research question that tested whether social exclusion indeed evoked subjective feelings of fear and psychological, as opposed to physical, restraint. In terms of the fear component, we found that social exclusion induced higher feelings of fear in comparison to social inclusion. This finding is consistent with the fear hypothesis (Gallup, 1977) and other models of fear-responses (Ratner, 1976; Schauer & Elbert, 2010). In terms of the psychological restraint component, it was shown that excluded participants reported subjective feelings of psychological restraint and threatened control (e.g., “I felt I was unable to significantly alter the event” and “I felt restrained during the game”). This suggests that, although participants were not physically restrained or withheld, they felt unable to influence their inclusion status (“I felt I was unable to influence the actions of others”). During the exclusion game, some participants clicked on the icons of other players or pressed several keys on the keyboard. However, these actions did not change the course of the game, as the behavior of the other players was pre-programmed. As such, the control and power of the participants was presumably threatened, as previous studies have shown (e.g., Williams et al., 2000; Zadro et al., 2004).

Overall, these findings are relevant because they give new insight into a possible explanation for the presence of trauma-like responses during and after relational aggression. Research with PTSD patients has shown that Ti can predict PTSD symptoms (Rocha-Rego et al., 2009) and might have a more negative impact on PTSD development than peritraumatic panic (Lima et al., 2010). The psychological restraint component is also highlighted in other areas of psychopathology. For instance, it has been demonstrated that psychological restraint is related to the development of several disorders, including social anxiety disorder (Taylor, Gooding, Wood, & Tarrier, 2011) and psychotic disorders (Schreier et al., 2009). Also, it is assumed that the experience of immobility and restraint in a psychological way, can serve as a mediating factor in the development and maintenance of psychiatric disorders. Recently, for instance, it was found that restraint mediated the relation between self-appraisals and suicidal behavior in patients with PTSD (Panagioti, Gooding, Taylor, & Tarrier, 2012). In summary, these studies suggest that subjective appraisals of restraint might be just as important for the emotional or behavioral consequences, as the physical actions during the event. Future research could investigate whether subjective appraisals of restraint are also important in non-interpersonal trauma.

Several limitations to the present study and suggestions for future research are outlined. One limitation is that the current sample only consists of healthy, female participants. Whether our results translate to bullying victims is therefore not known. Further, the present study demonstrated that particular subjective symptoms of Ti and psychological restraint occur during social exclusion but whether Ti also predicts PTSD symptoms in victims of relational aggression remains unclear.

Also, the physiological symptoms of Ti need further exploration. In the current study, the measure of average heart rate was probably a limited measure for physiological Ti. In order to conclude that social exclusion is related to physiological Ti, more sophisticated physiological measurements, such as bodily sway, should be included in future studies. Our final limitation is that this study did not include any behavioral or physical repeated outcome measures during exclusion that could track changes in immobility reactions. Therefore, the study could not examine the possibility that feelings of immobility were a side effect of not being able to perform any actions towards the other players. However, immobility measured with the TIS did not only cover the inability to perform actions. It also measured other symptoms of Ti such as eye closure, trembling, feeling fearful, cold and ashamed. It is difficult to explain these features as a “side effect” of not being able to perform actions. Future studies could improve the ecological validity of the manipulation by including verbal or non-verbal contact between participants and excluders. This could also improve the credibility of the Cyberball game since not all participants in our study believed they were playing with other students. On the contrary, being excluded by real people is not always necessary to induce feelings of exclusion as it has been shown that exclusion by a computer is also sufficient to induce feelings of exclusion (Zadro et al., 2004). A recent alternative paradigm that future studies could use is the new paradigm “O'cam,” which is more ecologically valid than the Cyberball game as it contains a Web-based interaction between the participant and (pre-recorded) excluders (Goodacre & Zadro, 2010). Because relational bullying is likely to be an ongoing and repetitive process (Olweus, 1991; Roland, 1989), the manipulation of social exclusion could also be varied in intensity and over time.

In conclusion, this study found that the subjective experience of Ti, including subjective feelings of fear and a threatened sense of control, can occur during social exclusion. This finding is highly important because it provides a new perspective on Ti as a fear-response and the circumstances in which Ti can occur. Whereas previous studies have only highlighted Ti-responses during situations of physical aggression, with high levels of fear and physical restraint, the present study demonstrates that events with relational aggression could also induce symptoms of Ti. In addition, the presence of Ti symptoms and psychological restraint might be important mechanisms to consider in explaining PTSD symptoms in victims of bullying who have suffered relational aggression, even in the absence of physical aggression. However, this hypothesis needs further testing in studies that specifically focus on these factors in a targeted group of victims of relational aggression, in combination with extended measurements of physiological Ti and psychological restraint.
